# Selection strategy for sedation depth in critically ill patients on mechanical ventilation

**DOI:** 10.1186/s12911-021-01452-7

**Published:** 2021-07-30

**Authors:** Longxiang Su, Chun Liu, Fengxiang Chang, Bo Tang, Lin Han, Huizhen Jiang, Weiguo Zhu, Na Hong, Xiang Zhou, Yun Long

**Affiliations:** 1 Department of Critical Care Medicine, State Key Laboratory of Complex Severe and Rare Diseases, Peking Union Medical College Hospital, Peking Union Medical College & Chinese Academy of Medical Sciences, 1 Shuaifuyuan, Dongcheng District, Beijing, 100730 China; 2 Digital Health China Technologies Co. Ltd., Floor 19, China Technology Exchange Building, 66 West Beisihuan Road, Haidian District, Beijing, 100080 China; 3 Information Center Department, State Key Laboratory of Complex Severe and Rare Diseases, Peking Union Medical College Hospital, Peking Union Medical College & Chinese Academy of Medical Sciences, Beijing, China; 4 Information Center Department/Department of Information Management, State Key Laboratory of Complex Severe and Rare Diseases, Peking Union Medical College Hospital, Peking Union Medical College & Chinese Academy of Medical Sciences, Beijing, China

**Keywords:** Mechanical ventilation, Clustering, Sedation and analgesia, Latent profile analysis, ICU

## Abstract

**Background:**

Analgesia and sedation therapy are commonly used for critically ill patients, especially mechanically ventilated patients. From the initial nonsedation programs to deep sedation and then to on-demand sedation, the understanding of sedation therapy continues to deepen. However, according to different patient’s condition, understanding the individual patient’s depth of sedation needs remains unclear.

**Methods:**

The public open source critical illness database Medical Information Mart for Intensive Care III was used in this study. Latent profile analysis was used as a clustering method to classify mechanically ventilated patients based on 36 variables. Principal component analysis dimensionality reduction was used to select the most influential variables. The ROC curve was used to evaluate the classification accuracy of the model.

**Results:**

Based on 36 characteristic variables, we divided patients undergoing mechanical ventilation and sedation and analgesia into two categories with different mortality rates, then further reduced the dimensionality of the data and obtained the 9 variables that had the greatest impact on classification, most of which were ventilator parameters. According to the Richmond-ASS scores, the two phenotypes of patients had different degrees of sedation and analgesia, and the corresponding ventilator parameters were also significantly different. We divided the validation cohort into three different levels of sedation, revealing that patients with high ventilator conditions needed a deeper level of sedation, while patients with low ventilator conditions required reduction in the depth of sedation as soon as possible to promote recovery and avoid reinjury.

**Conclusion:**

Through latent profile analysis and dimensionality reduction, we divided patients treated with mechanical ventilation and sedation and analgesia into two categories with different mortalities and obtained 9 variables that had the greatest impact on classification, which revealed that the depth of sedation was limited by the condition of the respiratory system.

## Background

The use and development of critical care medicine aims to provide comprehensive and effective life support for nonterminal critical patients with multiple organ dysfunction to save the patients’ lives and recover their quality of life to the greatest extent. After entering the ICU, the patients suffer obvious discomfort and pain due to the disease itself, such as hypoxia, shock, high fever, and surgery. In addition, since they lack a complete understanding of the disease status, treatment plan and prognosis, coupled with the various examinations, treatment measures and noisy medical environments, patients in the ICU can easily become anxious, irritable, painful and even delirious. This state not only causes tremendous pressure on the patient’s mental state but also leads to changes in the patient’s physiological state, even increasing the burden of related organ functions and worsening the condition in severe cases.

Analgesia and sedation therapy refer specifically to the application of medications to eliminate pain, relieve anxiety and restlessness, hypnotize and induce antegrade amnesia. Timely and dynamic assessments of the patients' anxiety, pain, and delirium, the provision of appropriate treatment based on disease state and diagnosis, and the delivery of appropriate analgesic and sedative medications have become the cornerstone for the smooth implementation of all other treatments in the ICU [1–3].

Currently recognized sedation treatment plans are based on analgesia; that is, the pain is evaluated first, and after appropriate treatment, a decision is made regarding the use of sedative drugs according to the patient’s needs.

However, Shehabi et al. found that many patients were deeply sedated within 48 h after entering the ICU, which constitutes an independent risk factor for prolonged mechanical ventilation and increased mortality in mechanically ventilated patients [4, 5]. Therefore, providing deep sedation to mechanically ventilated patients without evaluation and analysis, whether or not it is early in the patient’s care, may worsen the patient's condition, which of course we cannot see clearly.

For patients with severe lung disease, such as acute respiratory distress syndrome (ARDS), or spontaneously deep breathing, which leads to an increase in transpulmonary pressure, leading to lung injury, it is also unreasonable to administer light sedation. Instead, deeper sedation may be required to control the drastic changes in transpulmonary pressure to better protect the lungs.

Judging from the historical evolution of analgesia and sedation, the general consensus is to give on-demand sedation treatment to patients on mechanical ventilation in the ICU. From the initial plan involving no sedation to deep sedation and then to on-demand sedation, the understanding of sedation therapy continues to deepen. However, according to different patient’s condition, understanding the individual patient’s depth of sedation needs remains unclear. In this study, we used machine learning methods to identify patients who had received mechanical ventilation and undergone analgesia and sedation treatment into clinical subtypes and analyzed clinical features that affect the classification in an attempt to find a standard that determines the depth of sedation treatment needs for the patients.

## Methods

**Fig. 1:**
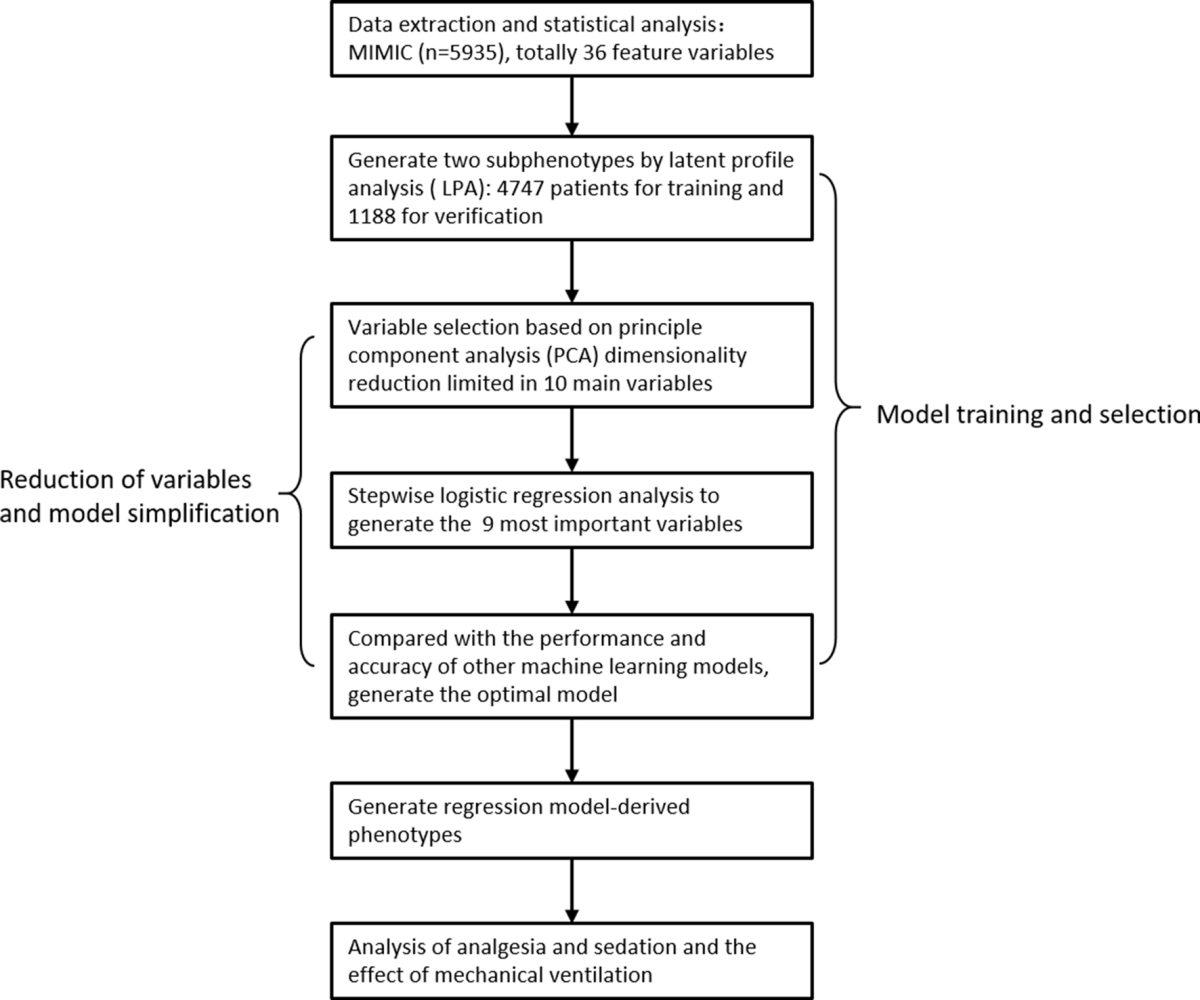
Overview of the primary analysis plan

An overview of the primary analysis plan is outlined in Fig. 1. Briefly, latent profile analysis (LPA) was conducted on the Medical Information Mart for Intensive Care III (MIMIC III) dataset, and the resultant phenotypes based on 36 variables served as the standard for developing stepwise logistic regression variable selection. The dataset was split into a training dataset (80%) and a verification dataset (20%). To reduce the number of variables and simplify the model, principal component analysis (PCA) dimensionality reduction was used for selecting important variables. The most important variables were, in turn, used to develop stepwise logistic regression classifier models. The models were compared with other machine-learning models in terms of comprehensive model performance to verify the optimal model. The model-derived phenotypes were generated and analyzed.

### Dataset

The MIMIC-III dataset is a large, freely available database comprising deidentified health-related data associated with over forty thousand patients who stayed in the critical care units of the Beth Israel Deaconess Medical Center between 2001 and 2012. The database includes information such as demographics, vital sign measurements made at the bedside, laboratory test results, procedures, medications, caregiver notes, imaging reports, and mortality [6, 7].

This study used the data from a total of 5935 adults from the MIMIC III database who had received mechanical ventilation and were administered sedative analgesics during their ICU stay. The enrolled patients’ data were extracted from the MIMIC-III database according to the following criteria:

1. Mechanically ventilated patients: patients with a non-null value for the positive expiratory end pressure (PEEP) were included.
2. Use of sedative and analgesic drugs: Considering the real data available in the database and clinical experiences, we selected five sedative or analgesic drugs, fentanyl, midazolam, morphine, propofol, and dexmedetomidine. Patients who had used one of them (that is, the corresponding dose value was not empty) were included in the study cohort.

### Statistical methods

In total of 36 candidate feature variables associated with mechanical ventilation were selected for statistical analysis, as shown in Table 1.

**Table 1:** Thirty-six feature variables and classifications related to mechanical ventilation

Demographic characteristics and scores	Age, SAPS II, SOFA scores, Richmond-ASS scores
Laboratory values	White blood cell (WBC), Hemoglobin (Hb), Hematocrit, Platelet, Urea Nitrogen (BUN), pH, pO_2_, pCO_2_, Bicarbonate (HCO_3_−), Lactate (Lac), Potassium (K), Sodium (Na), Chlorine (Cl), Total calcium (TotalCa), Partial thromboplastin time (PTT)
Persistent variables	Fluid balance
Life support equipment variables	Positive expiratory end pressure (PEEP), Oxygen concentration in the inhalation gas (FiO_2_), Tidal volume (VT), Peak airway pressure (Ppeak), Mean airway pressure (Pmean), Platform pressure (Pplat), Respiratory rate (RR)
Monitoring data	Heart rate (HR), Mean arterial pressure (MAP), Temperature, Pulse oxygen saturation (SpO_2_%)
Sedative and analgesic drugs	Dexmedetomidine, Fentanyl, Midazolam, Morphine, Propofol

For each variable, the average value over the first 24 h after analgesia and sedation administration was calculated and used; the exceptions were total calcium, fluid balance and sedative and analgesic drugs, for which the total amount during the first day was used. To explore the subphenotypes of patients given analgesia and sedation, we first evaluated the distributions, absence and correlations of candidate feature variables.

For the preprocessing of missing values and outliers, given the scarcity of samples, we did not simply adopt variable removal and mean replacement. Instead, we used chain equations for multiple imputation for missing data [8] and logarithmic transformation. For the sedative and analgesic drugs, we filled null values with 0 according to the actual situation. In the correlation evaluation, we used the ranking statistics from sensitivity analysis to exclude highly correlated variables.

### Latent profile analysis (LPA)

We used the unsupervised clustering method commonly used in medical research in our study. Consensus clustering [9] and latent profile analysis (LPA) were used within a Gaussian mixture model. Latent profile analysis is a probabilistic or model-based technique that is a variant of traditional cluster analysis [10, 11].

To better visualize the results, multiple types of graphs were used for analysis and display: (1) Box plots were used to show the difference in phenotypes through the means and standard deviations of the variables; (2) T-distributed stochastic neighbor embedding (t-SNE) was used to reduce multidimensional variables to two dimensions to visualize the subphenotypes; and (3) survival analysis curves were used to demonstrate the 28-day survival curve of each phenotype.

To describe the potential relationship between the analgesia and sedation phenotypes and the 36 features, we also compared the means, standard deviations and proportion of each phenotype for these characteristics. At the same time, we performed the χ^2^ test on the cumulative mortality of each phenotype at 28 days to determine whether there were significant differences.

### Variable selection based on principal component analysis (PCA)

Principal component analysis (PCA) is a common data analysis method that is often used for dimensionality reduction of high-dimensional data and can be used to extract the main feature components of the data. In this study, we applied the PCA dimensionality reduction method to find and verify the variables that had the greatest impact on the effect of model classification, which may guide clinical application in the future. First, the original feature set was transformed by PCA, and then the weights of the original features in the transformation were obtained by analyzing the transformation matrix. The final feature subset was selected according to the order of the weights from high to low. To limit the complexity of our model, the ten most important variables were chosen for the next step in the model analysis.

### Logistic regression model

The top ten variables identified by PCA were used in forward stepwise regression using the dataset. Logistic regression models of increasing complexity were generated by sequential addition of variables. The order in which the variables were entered into the model was determined by the findings of the stepwise regression analysis. Model performance was assessed by generating receiver operating characteristic (ROC) curves and calculating the area under the ROC curve (AUROC).

To confirm the advancement of our model, we compared the performance of the logistic regression model and other classifiers, such as XGBoost, random forest, and decision tree. Accuracy, precision, recall, F1-score and AUC (area under curve) were used to evaluate the performance of the classifiers.

### Analysis of analgesia and sedation and the effect of mechanical ventilation

The Richmond-ASS [12] reflects the degree of sedation of the patient. The lower the score, the better the sedation effect is. Based on previous clinical studies and experiences, we divided patients into three categories with different degrees of sedation according to the Richmond-ASS: [− 5, − 3], (− 3, 0], and (0, 3]. It is generally considered that patients with 0 points and below have higher levels of sedation. In Richmond-ASS table, 4 is the highest score which stands for combative. Among our patients included in the cohort, the highest score was 3, so the patients who were unsuccessfully sedated are classified as (0, 3].

## Results

### Statistical description of patient information

We included data from a total of 5,935 patients who had received mechanical ventilation during their ICU stay and extracted their feature data from within 24 h after admission to the ICU. Data from 4747 patients were selected as the training set, and data from 1188 patients were selected as the validation set. Table 2 shows the statistical description of the two data sets.

**Table 2:** Statistical description of the training set and validation set in the study

Features (mean ± SD)	Training set (n = 4747)	Validation set (n = 1188)
Age	63.83 ± 16.37	63.22 ± 16.29
SAPS II	40.21 ± 15.04	39.56 ± 14.85
SOFA scores	5.49 ± 3.53	5.36 ± 3.48
White blood cell (WBC)	12.52 ± 8.98	12.44 ± 6.27
Hemoglobin (Hb)	10.67 ± 1.90	10.74 ± 1.93
Hematocrit	31.91 ± 5.51	32.07 ± 5.48
Platelet	210.77 ± 109.94	210.00 ± 108.69
Urea nitrogen (BUN)	25.31 ± 20.39	24.95 ± 20.04
pH	7.37 ± 0.07	7.37 ± 0.07
pO_2_	189.14 ± 85.98	191.54 ± 89.92
pCO_2_	41.91 ± 8.66	41.53 ± 8.06
Bicarbonate (HCO_3_−)	23.62 ± 4.21	23.57 ± 4.01
Lactate (Lac)	2.23 ± 1.45	2.26 ± 1.52
Potassium (K)	4.22 ± 0.54	4.22 ± 0.57
Sodium (Na)	138.17 ± 4.04	138.17 ± 4.16
Chlorine (Cl)	105.33 ± 5.29	105.28 ± 5.37
Total calcium (TotalCa)	8.24 ± 0.74	8.23 ± 0.73
Partial thromboplastin time (PTT)	35.81 ± 15.29	36.14 ± 15.82
Fluid balance	4580.69 ± 9379.25	4687.70 ± 4268.08
Positive expiratory end pressure (PEEP)	5.99 ± 2.01	5.87 ± 1.90
Oxygen concentration in the inhalation gas (FiO_2_)	56.06 ± 13.29	55.37 ± 12.74
Tidal volume (VT)	520.06 ± 178.15	517.18 ± 159.60
Peak airway pressure (Ppeak)	20.28 ± 5.62	20.36 ± 5.50
Mean airway pressure (Pmean)	9.45 ± 2.85	9.40 ± 2.77
Platform pressure (Pplat)	18.29 ± 4.18	18.23 ± 3.97
Heart rate (HR)	86.06 ± 15.41	86.05 ± 15.63
Mean arterial pressure (MAP)	78.35 ± 11.68	78.42 ± 11.28
Respiratory rate (RR)	18.76 ± 3.71	18.62 ± 3.85
Temperature	36.83 ± 0.68	36.84 ± 0.67
Pulse oxygen saturation (SpO_2_%)	97.55 ± 2.26	97.61 ± 2.19
Richmond-ASS scores	− 1.59 ± 1.49	− 1.59 ± 1.55
Dexmedetomidine	0.12 ± 0.77	0.12 ± 0.64
Fentanyl	3.52 ± 11.43	3.12 ± 9.97
Midazolam	65.45 ± 296.75	55.62 ± 208.40
Morphine	15.23 ± 84.53	13.07 ± 38.04
Propofol	3929.18 ± 8581.78	4066.48 ± 9464.44

### Clustering for clinical phenotypes

**Fig. 2:**
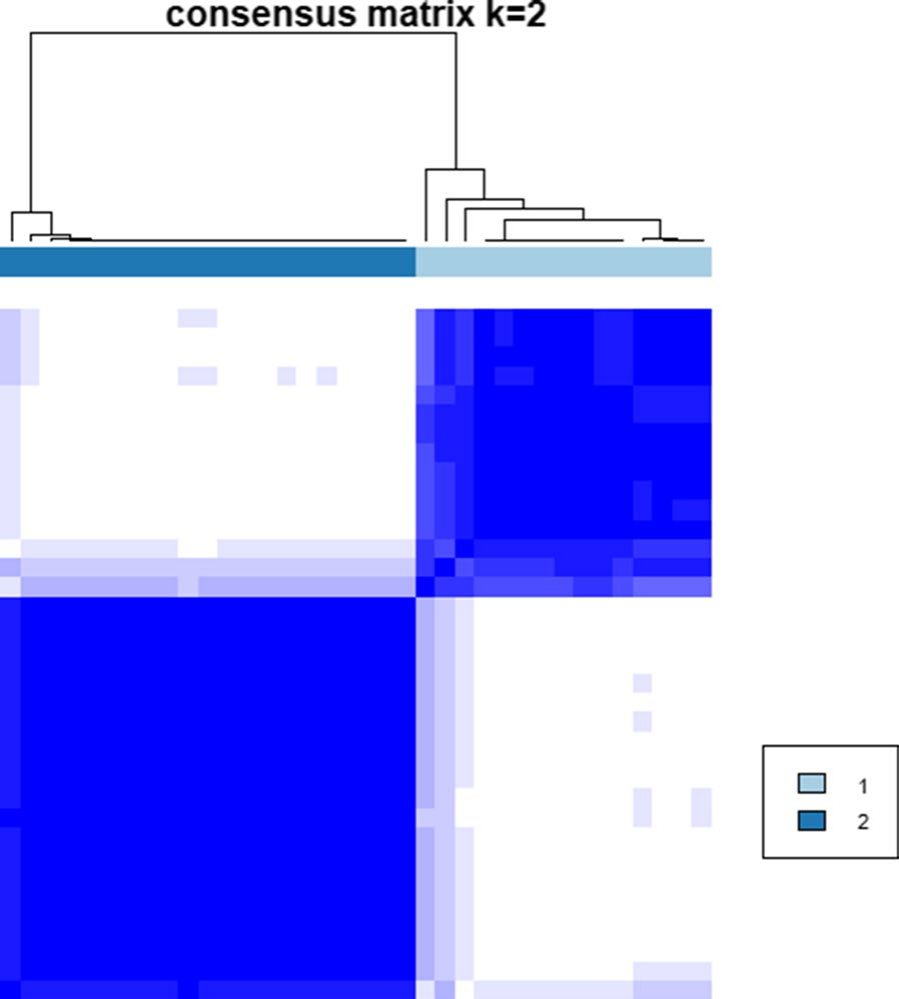
Matrix heatmap (k = 2) shows that clustering into 2 categories is the best fit

After data preprocessing, we used consensus clustering to classify 2–10 phenotypes to confirm the optimal number of phenotypes. According to Fig. 2, we found that dividing the data into two phenotypes was the best fit.

**Fig. 3:**
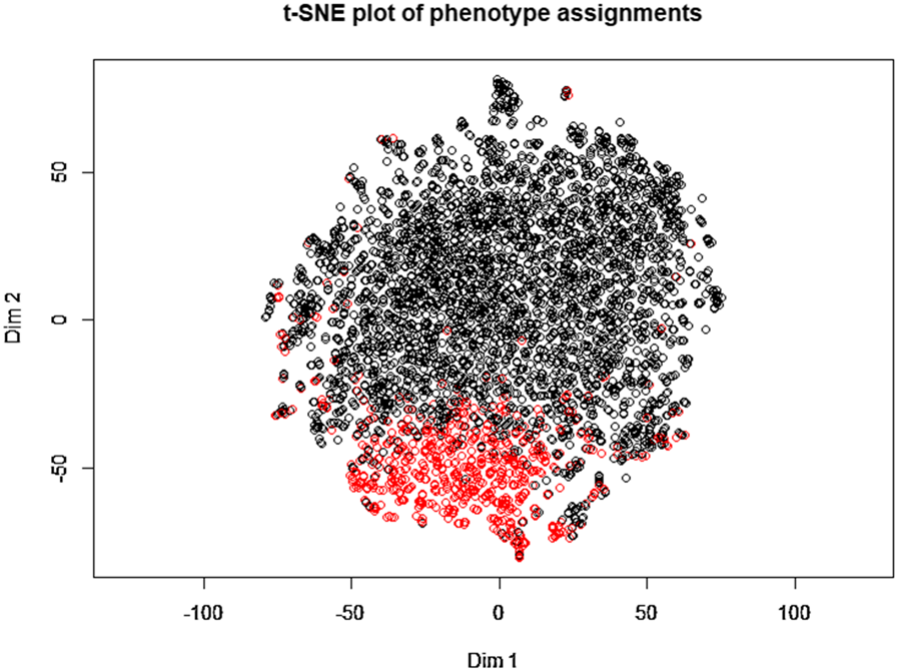
t-SNE plot of phenotype assignments

Considering that the data types of the candidate feature variables were all numerical, we then used the Gaussian mixture model to perform potential profile analysis [13] to cluster the data into two phenotypes. Figure 3 shows the two-dimensional clustering status shown by the t-SNE diagram.

**Fig. 4:**
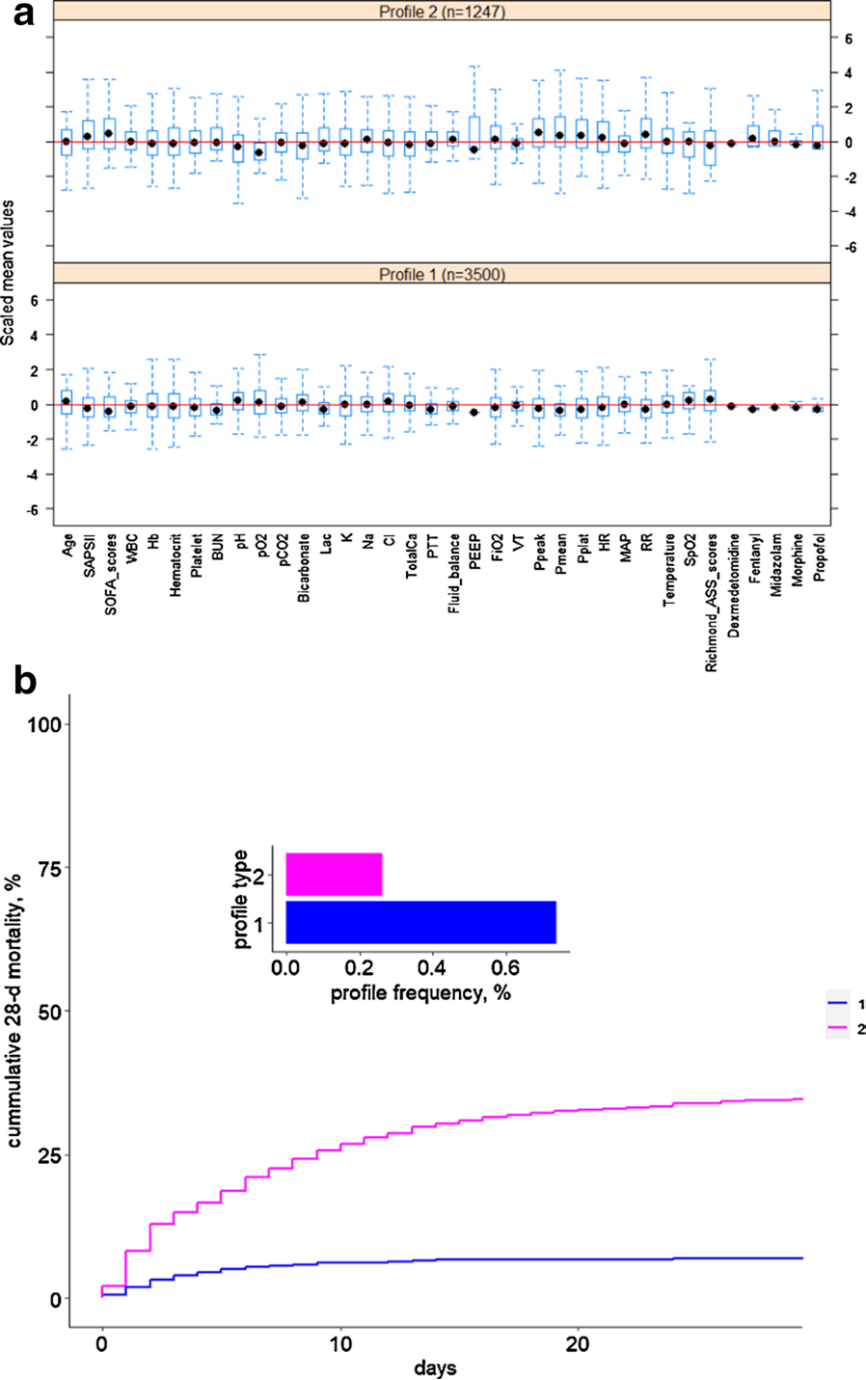
The box plot (**a**) and 28-day survival curve (**b**) of the two phenotypes obtained with the 36 variables in the training set

**Fig. 5:**
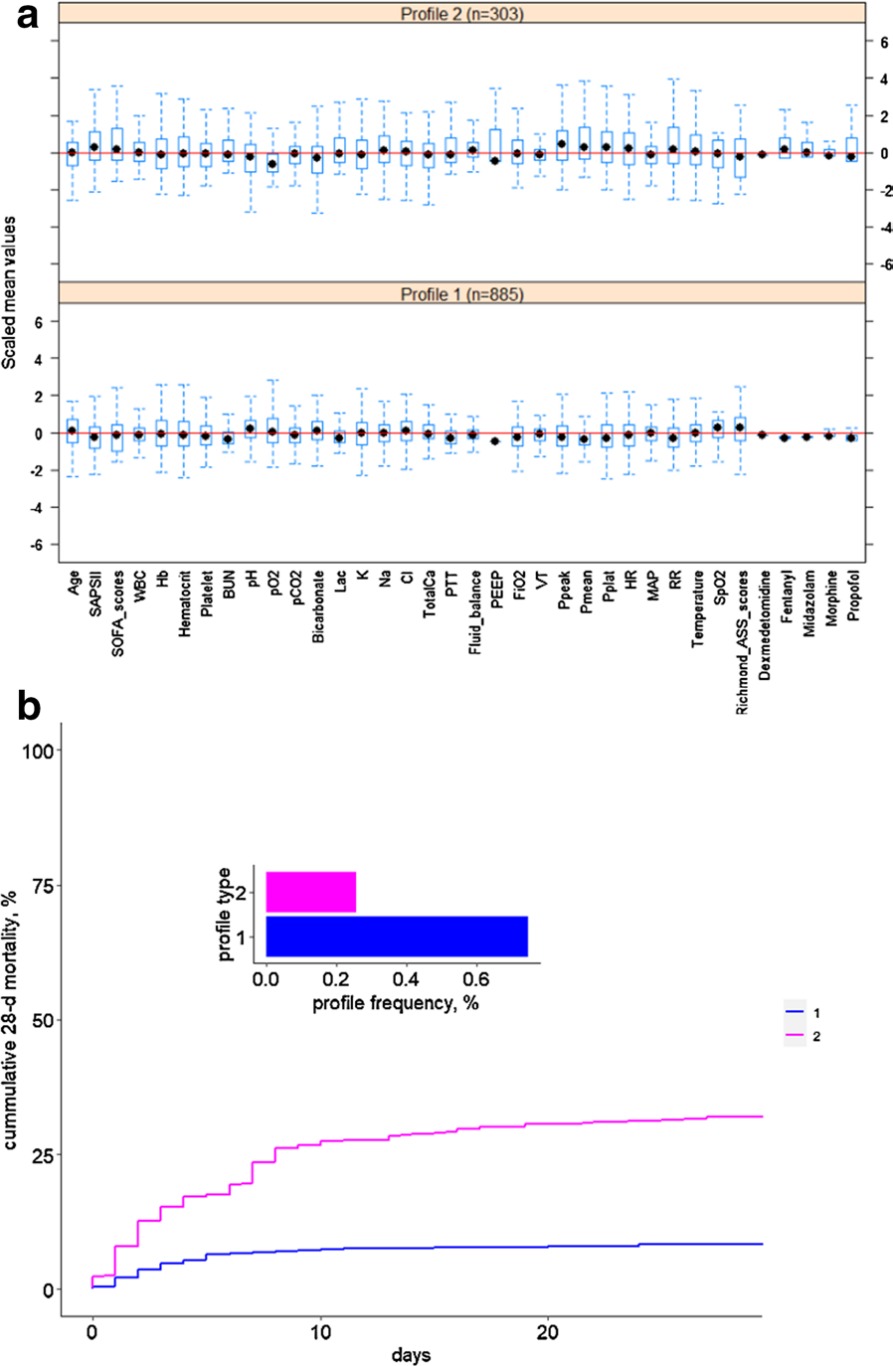
The box plot (**a**) and 28-day survival curve (**b**) of two phenotypes obtained with the 36 variables in the validation set

As shown in Fig. 4a and Table 3, the variables were scaled for each phenotype. Broad differences were observed in the distributions of the scaled variables across phenotypes. Of the 36 variables measured, 29 were significantly different across phenotypes in the training set with *P* < 0.05. Figure 4b shows the 28-day survival curves of the two phenotypes in the training set. The validation set shows the same trend (Fig. 5).

**Table 3:** Statistical description of the two phenotypes of the training set

Features (mean ± SD)	Profile1 (n = 3500)	Profile2 (n = 1247)	*P* value
Age	64.57 ± 16.19	61.77 ± 16.70	< 0.001
SAPS II	37.92 ± 13.47	46.63 ± 17.22	< 0.001
SOFA scores	4.88 ± 3.00	7.20 ± 4.28	< 0.001
White blood cell (WBC)	11.78 ± 4.64	14.59 ± 15.52	< 0.001
Hemoglobin (Hb)	10.65 ± 1.81	10.75 ± 2.13	0.122
Hematocrit	31.77 ± 5.18	32.28 ± 6.33	0.005
Platelet	207.19 ± 101.90	220.82 ± 129.40	< 0.001
Urea Nitrogen (BUN)	22.49 ± 15.99	33.20 ± 27.95	< 0.001
pH	7.38 ± 0.06	7.34 ± 0.09	< 0.001
pO_2_	203.65 ± 85.69	148.41 ± 72.72	< 0.001
pCO_2_	41.49 ± 7.34	43.08 ± 11.52	< 0.001
Bicarbonate (HCO_3_−)	23.95 ± 3.64	22.67 ± 5.41	< 0.001
Lactate (Lac)	2.01 ± 0.93	2.85 ± 2.25	< 0.001
Potassium (K)	4.21 ± 0.51	4.26 ± 0.62	0.003
Sodium (Na)	138.13 ± 3.65	138.28 ± 4.96	0.245
Chlorine (Cl)	105.49 ± 4.88	104.88 ± 6.29	0.001
Total calcium (TotalCa)	8.27 ± 0.63	8.18 ± 0.99	< 0.001
Partial thromboplastin time (PTT)	34.06 ± 12.31	40.74 ± 20.78	< 0.001
Fluid balance	4023.84 ± 3505.98	6143.63 ± 17,240.77	< 0.001
Positive expiratory end pressure (PEEP)	5.56 ± 1.24	7.20 ± 3.01	< 0.001
Oxygen concentration in the inhalation gas (FiO_2_)	54.52 ± 11.58	60.39 ± 16.45	< 0.001
Tidal volume (VT)	504.45 ± 91.65	563.87 ± 307.72	< 0.001
Peak airway pressure (Ppeak)	19.21 ± 4.72	23.29 ± 6.73	< 0.001
Mean airway pressure (Pmean)	8.76 ± 1.88	11.40 ± 3.99	< 0.001
Platform pressure (Pplat)	17.53 ± 3.43	20.44 ± 5.22	< 0.001
Heart rate (HR)	84.46 ± 13.91	90.54 ± 18.27	< 0.001
Mean arterial pressure (MAP)	78.46	78.05 ± 17.84	0.288
Respiratory rate (RR)	18.04 ± 3.13	20.79 ± 4.40	< 0.001
Temperature	36.84 ± 0.58	36.79 ± 0.92	0.037
Pulse oxygen saturation (SpO_2_%)	97.84 ± 1.54	96.72 ± 3.43	< 0.001
Richmond-ASS scores	− 1.39 ± 1.34	− 2.15 ± 1.74	< 0.001
Dexmedetomidine	0.01 ± 0.05	0.42 ± 1.47	< 0.001
Fentanyl	0.57 ± 1.26	11.82 ± 19.99	< 0.001
Midazolam	8.29 ± 23.13	225.89 ± 546.80	< 0.001
Morphine	7.27 ± 12.10	37.56 ± 161.65	< 0.001
Propofol	2219.76 ± 3271.76	8727.05 ± 14,805.89	< 0.001

### The most influential variables according to PCA

**Fig. 6:**
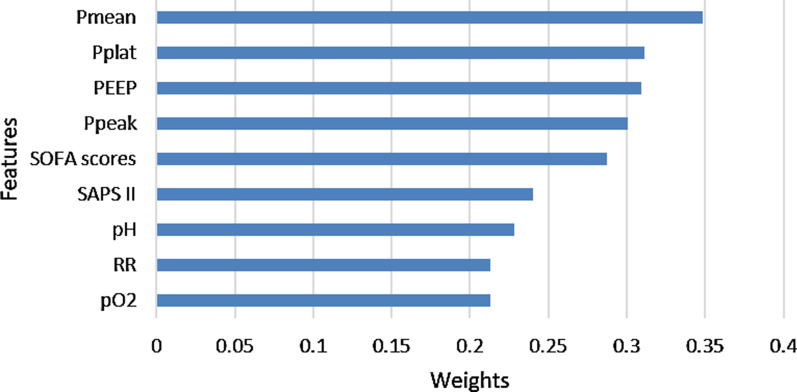
The 9 most important variables identified by PCA

The 10 most important variables obtained from PCA are presented in Fig. 6. They include Pmean, Pplat, PEEP, Ppeak, SOFA score, SAPS II, pH, respiratory rate (RR), FiO_2_, pO_2_.

**Fig. 7:**
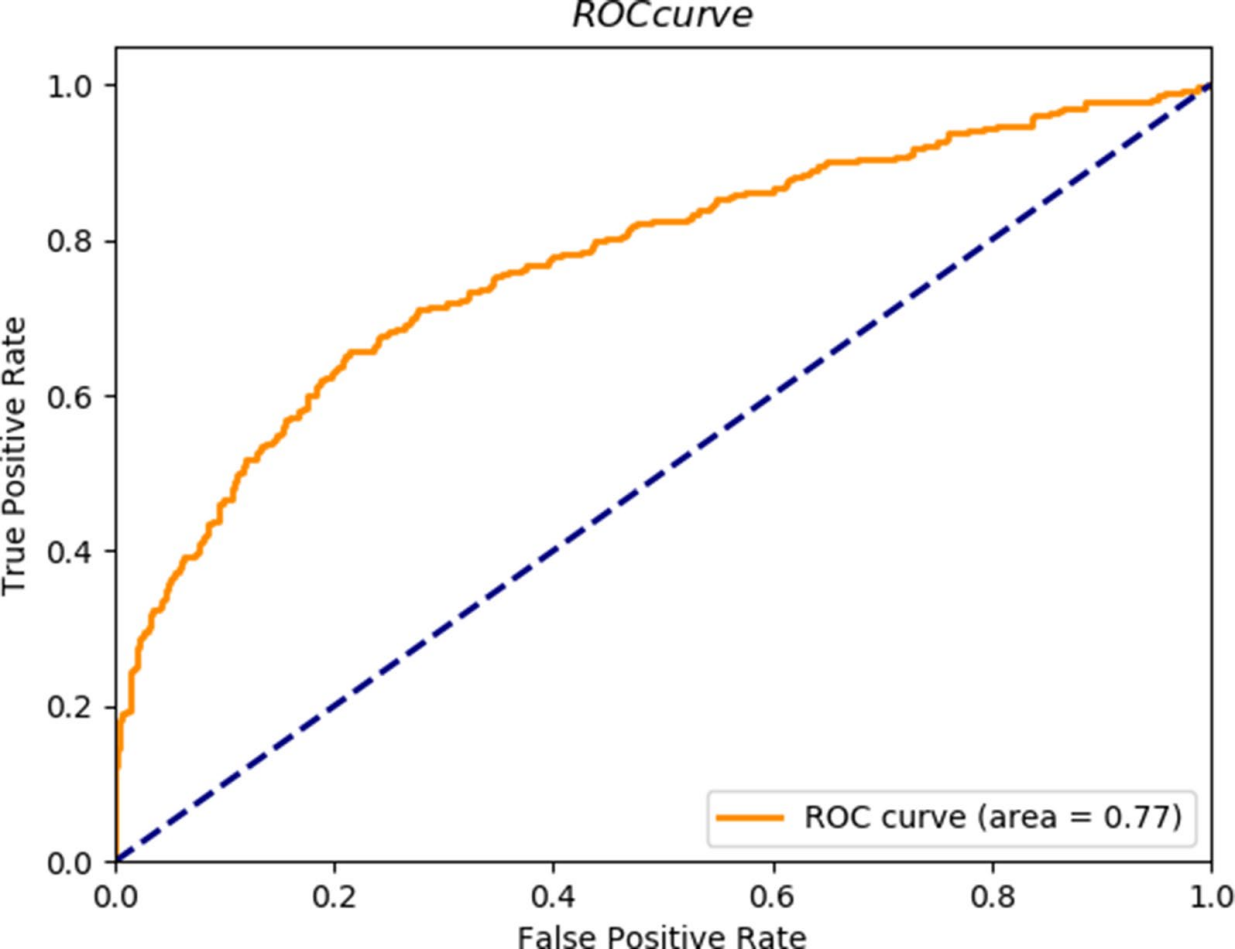
ROC curve of the 9-variable model

Forward stepwise regression did not eliminate any of the ten variables. Increasing the model complexity via the sequential addition of variables led to improved model performance. From Table 4, we can see that there was a relative plateauing by the time the 9-variable and 10-variable models were created. Considering the best balance between classification accuracy and model simplicity, the 9-variable model was defined as the best classifier model in our research. Figure 7 shows the receiver operating characteristic (ROC) curve of the 9-variable model, and the area under the curve (AUC) reached 0.77.

**Table 4:** Logistic regression model composition and accuracy with the dataset

	Pmean	Pplat	PEEP	Ppeak	SOFA scores	SAPS II	pH	RR	pO2	FiO2	AUC
Model1	Yes										0.70
Model2	Yes	Yes									0.70
Model3	Yes	Yes	Yes								0.70
Model4	Yes	Yes	Yes	Yes							0.70
Model5	Yes	Yes	Yes	Yes	Yes						0.72
Model6	Yes	Yes	Yes	Yes	Yes	Yes					0.73
Model7	Yes	Yes	Yes	Yes	Yes	Yes	Yes				0.74
Model8	Yes	Yes	Yes	Yes	Yes	Yes	Yes	Yes			0.75
Model9	Yes	Yes	Yes	Yes	Yes	Yes	Yes	Yes	Yes		0.77
Model10	Yes	Yes	Yes	Yes	Yes	Yes	Yes	Yes	Yes	Yes	0.77

To confirm the advancement of our model, we compared its performance with that of XGBoost, random forest and decision tree in terms of several parameters, such as accuracy, precision, recall, F1-score and AUC. As Table 5 shows, random forest has the highest precision with 0.7947, and XGBoost has the highest recall with 0.6574, but our logistic regression model has the highest accuracy, F1-score, and AUC with 0.7997, 0.6771, and 0.7725, respectively. Thus, we believe the logistic regression model performed better than other classifiers.

**Table 5:** Performance of different classifiers

Classifier	Accuracy	Precision	Recall	F1-score	AUC
Logistic regression	0.7997	0.7625	0.6550	0.6771	0.7725
XGBoost	0.7870	0.7245	0.6574	0.6753	0.7464
Random forest	0.7921	0.7947	0.6130	0.6265	0.7684
Decision tree	0.7012	0.6168	0.6247	0.6200	0.6247

**Fig. 8:**
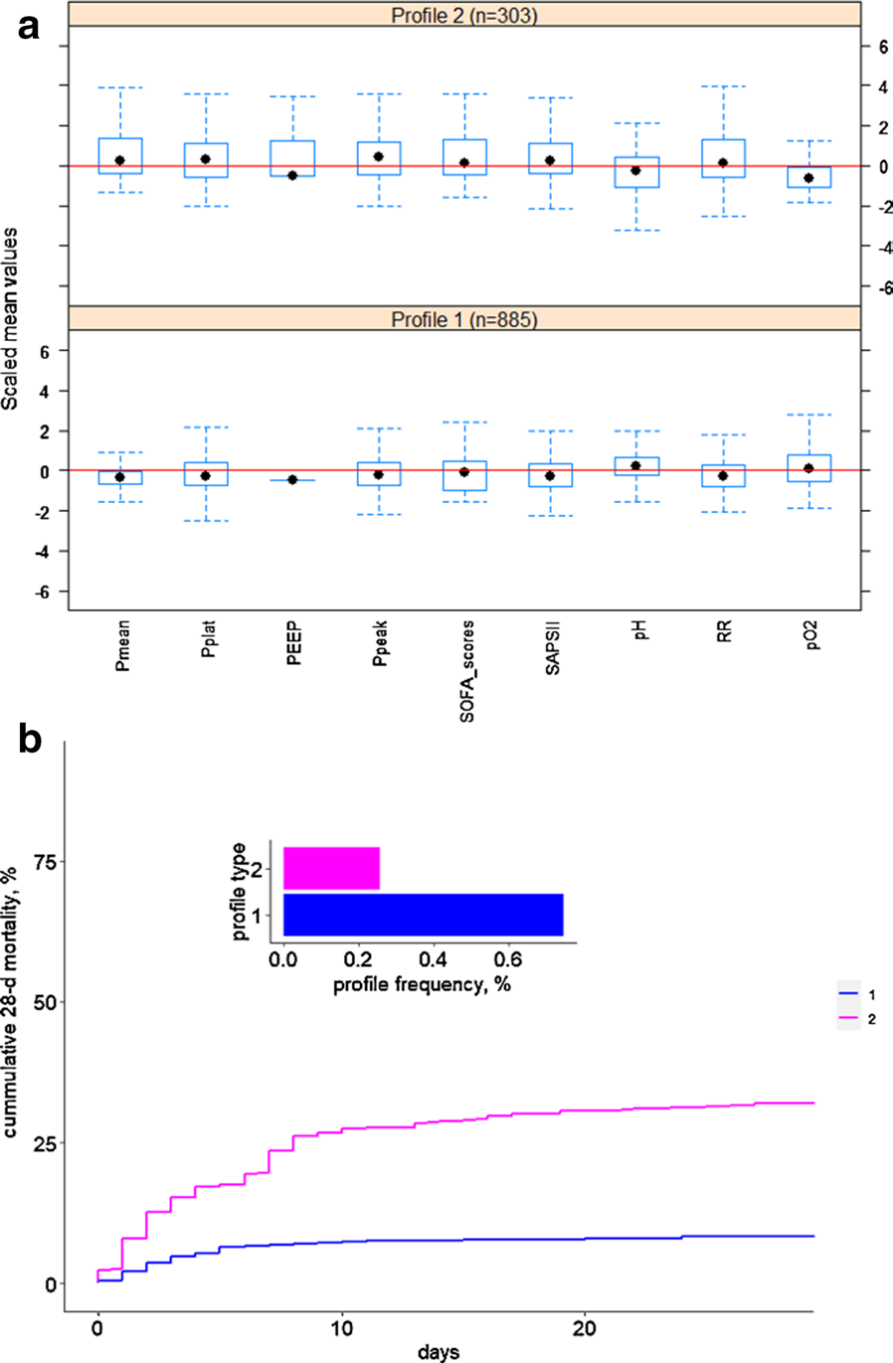
The box plot (**a**) and 28-day survival curve (**b**) of the two phenotypes obtained with the 9 most important variables in the validation set

Based on the 9 most influential variables obtained by PCA, we also obtained two phenotypes, and the corresponding box plot and the 28-day survival curve of the validation set are shown in Fig. 8. The 9 variables were significantly different between the two phenotypes.

**Fig. 9:**
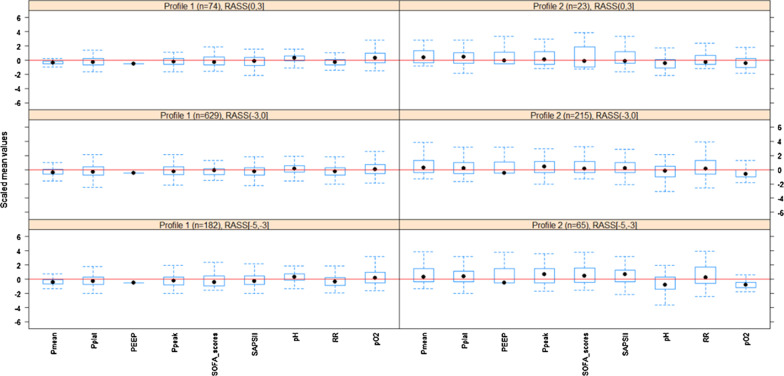
The validation set was further divided into three levels of sedation: deep sedation, light sedation and unsuccessful sedation. The corresponding RASS scores are [− 5 − 3], (− 3 0] and (0 3]

To further explore the relationship between different phenotypes and the degree of sedation and analgesia, the data of the two phenotypes from the validation set were then divided into three different levels of sedation according to the Richmond-ASS score, where [− 5, − 3] stands for deep sedation, (− 3, 0] stands for light sedation and (0, 3] stands for unsuccessful sedation treatment, as shown in Fig. 9. From this figure, Profile 1 accounted for 66.42% (n = 182) in the deep sedation [− 5, − 3] group, which indicated that these patients maybe reduce the depth of sedation. Profile 2 accounted for 25.47% (n = 215) in the light sedation (− 3, 0], which indicated that these patients need deeper sedation to meet their breathing needs. The proportion of unsuccessful sedation treatment (0, 3] patients is relatively small.

## Discussion

This study divided mechanically ventilated patients who were administered analgesia and sedation into two categories. By reducing the dimensionality of the PCA, we can see that the obvious difference between the two types lies in the ventilator-based parameters. This shows that sedation is necessary for mechanically ventilated patients, who need to be administered sedation therapy to assist in lung self-treatment. By dividing the validation cohort into three categories, the result showed that patients with higher ventilator conditions require deeper levels of sedation. If the patient’s respiratory system disease is improving, the patient can be identified in time according to the model. Patients with profile1 require reduction in the level of sedation earlier or transition to the awake state in time, and patients with profile2 may need to continue with a deeper sedation mode to continue treatment.

Mechanical ventilation remains the main indication for continuous sedation in the ICU. For example, given the severity of ARDS, the needs of patients with regard to analgesia and sedation will be substantially different. Yoshida et al. [14] conducted a controlled study of spontaneous breathing and muscle relaxation therapy by making animal models of acute experimental lung injury of different severities. The results showed that with mild lung injury, spontaneous respiration can maintain a lower peak airway pressure and lead to more obvious histological improvement, while with severe lung injury, a lower peak airway pressure can be maintained when there is no spontaneous respiration; that is, under muscle relaxation, lower peak pressure and better histological improvement can be achieved. Papazian et al. [15] also confirmed that for patients with severe ARDS, deep analgesia and sedation combined with muscle relaxants can improve their 90-day survival rate and shorten the time of mechanical ventilation and do not increase the incidence of muscle weakness. That is, for patients with severe ARDS, retaining spontaneous breathing and maintaining mild sedation may be harmful. The mechanism is related to the deep analgesia, sedation and muscle relaxation used to improve human–machine synchronization, reduce transpulmonary pressure, and reduce ventilator-related lung damage. However, as the condition of the ARDS patient improves, the continuous implementation of deep sedation strategies for patients with mild ARDS becomes more harmful. Shehabi et al. [16] suggested that the proportion of patients with excessive sedation within 48 h of mechanical ventilation in the ICU is as high as 68%. The disadvantages of long-term deep sedation are as follows: the patient's consciousness cannot be judged in time; it increases complications (such as muscle atrophy and weakness), ventilator dependence, venous thromboembolism, pressure ulcers and delirium; it inhibits circulation and gastrointestinal motility; it prolongs weaning time and length of ICU hospital stay; and it increases the risk of acquired ventilator-associated pneumonia (VAP). [17]. In particular, coma caused by sedative drugs may increase the mortality rate of ICU patients, prolong intubation time and ICU hospitalization time, and cause long-term neuropsychological dysfunction [18]. For ICU patients, shallow sedation can effectively prevent excessive sedation, ensure that the patient is comfortable, safe and sufficiently awake and responsive, make it easy to determine whether to wean and extubate, and assess pain, delirium, and the ability to carry out early activities [19]. By establishing this model, the dose ranges of sedative drugs can be determined according to the parameters of the ventilator used during treatment. In other words, for the patient, what kind of ventilator parameters require deep sedation or light sedation? In this way, mechanically ventilated patients with serious pulmonary disease were found to need appropriate sedation therapy, while for recovery after treatment, the degree of sedation can be reduced, in turn reducing the secondary harm of mechanical ventilation to the patients.

There are some limitations in this study. First, MIMIC III was built in 2012, and there was insufficient knowledge about analgesia and sedation at that time. Second, respiratory waveforms and other information were not available; thus, although the score reached the standard, it was not clear whether the ventilator still allowed spontaneous breathing after sedation (this would aggravate the injuries related to mechanical ventilation). Third, the effects of muscle relaxation on mechanical ventilation patients were not analyzed in this study. In the future, it may be necessary to conduct related research by incorporating other databases, such as MIMIC IV.

## Conclusion

The patients in the MIMIC III database who underwent mechanical ventilation therapy and took sedative and analgesic drugs during their ICU stay were selected as the research sample. Based on 36 variables, we applied latent profile analysis to divide these patients into two categories with different mortalities. To further identify the key variables that affected the classification, we applied dimensionality reduction to select the 9 most critical variables, most of which were parameters associated with mechanical ventilation. After that, we divided the patients into three subphenotypes, deep sedation, light sedation and unsuccessful sedation, according to the degree of sedation and analgesia and correlated them with the critical parameters of mechanical ventilation. That is, patients with profile1 were more suitable for reduced sedation, and patients with profile2 were more suitable for maintaining deeper sedation. We look forward to adjusting the parameters of mechanical ventilation according to the degree of sedation in the future to achieve fine individualized treatment in the ICU.

## Abbreviations

ICU: Intensive care unit
MIMIC III: Medical information mart for intensive care III
ARDS: Acute respiratory distress syndrome
LPA: Latent profile analysis
PCA: Principal component analysis
PEEP: Positive expiratory end pressure
t-SNE: T-distributed stochastic neighbor embedding
ROC: Receiver operating characteristic
AUC: Area under curve
AUROC: Area under the ROC curve
RR: Respiratory rate
SOFA score: Sequential organ failure assessment score
SAPS II: Simplified acute physiology score II
RASS: Richmond agitation-sedation scale
VAP: Ventilator-associated pneumonia
